# 4-Ethylphenol—fluxes, metabolism and excretion of a gut microbiome derived neuromodulator implicated in autism

**DOI:** 10.3389/fmolb.2023.1267754

**Published:** 2023-10-12

**Authors:** Francesca Day, Justin O’Sullivan, Chris Pook

**Affiliations:** ^1^ Liggins Institute, Waipapa Taumata Rau—The University of Auckland, Auckland, New Zealand; ^2^ The Maurice Wilkins Centre, The University of Auckland, Auckland, New Zealand; ^3^ MRC Lifecourse Epidemiology Unit, University of Southampton, Southampton, United Kingdom; ^4^ Australian Parkinson’s Mission, Garvan Institute of Medical Research, Sydney, NSW, Australia; ^5^ A*STAR Singapore Institute for Clinical Sciences, Singapore, Singapore

**Keywords:** 4-ethylphenol, 4-ethylphenol sulfate, autism, gut microbiome, neuromodulators, host microbiome modulation, uremic toxins, membrane integrity

## Abstract

Gut-microbiome-derived metabolites, such as 4-Ethylphenol [4EP], have been shown to modulate neurological health and function. Although the source of such metabolites is becoming better understood, knowledge gaps remain as to the mechanisms by which they enter host circulation, how they are transported in the body, how they are metabolised and excreted, and the way they exert their effects. High blood concentrations of host-modified 4EP, 4-ethylphenol sulfate [4EPS], are associated with an anxiety phenotype in autistic individuals. We have reviewed the existing literature and discuss mechanisms that are proposed to contribute influx from the gut microbiome, metabolism, and excretion of 4EP. We note that increased intestinal permeability is common in autistic individuals, potentially explaining increased flux of 4EP and/or 4EPS across the gut epithelium and the Blood Brain Barrier [BBB]. Similarly, kidney dysfunction, another complication observed in autistic individuals, impacts clearance of 4EP and its derivatives from circulation. Evidence indicates that accumulation of 4EPS in the brain of mice affects connectivity between subregions, particularly those linked to anxiety. However, we found no data on the presence or quantity of 4EP and/or 4EPS in human brains, irrespective of neurological status, likely due to challenges sampling this organ. We argue that the penetrative ability of 4EP is dependent on its form at the BBB and its physicochemical similarity to endogenous metabolites with dedicated active transport mechanisms across the BBB. We conclude that future research should focus on physical (e.g., ingestion of sorbents) or metabolic mechanisms (e.g., conversion to 4EP-glucuronide) that are capable of being used as interventions to reduce the flux of 4EP from the gut into the body, increase the efflux of 4EP and/or 4EPS from the brain, or increase excretion from the kidneys as a means of addressing the neurological impacts of 4EP.

## Introduction

Catabolism of the aromatic amino acids phenylalanine and tyrosine by certain gut microbiota yields 4-ethylphenol [4EP] (Hsiao et al., 2013; Needham et al., 2022; Zheng et al., 2021). Dietary and systemic availability of aromatic amino acids and the presence of microbiota with the functional capacity to create 4EP regulate its production in the body. Rates of production are important to health as plasma concentrations of 4EP and its more polar, sulfated form, 4-ethylphenol sulfate [4EPS], correlate with neurological changes in humans and mice (Hsiao et al., 2013; Needham et al., 2021; 2022; Velásquez-Jiménez et al., 2021). For example, a reduction of plasma 4EPS concentrations from 29 μg/L to 9 μg/L correlated with significant reductions in core autism-behaviours, such as anxiety, in autistic children (Campbell et al., 2022). Similarly, accumulation of 4EPS in the mouse brain increased degradation of neuronal axon myelin sheath; the insulator of the neurons (Needham et al., 2022).

Autism is a spectrum of complex neurological phenotypes that alter how autistic people perceive the world, think and behave, communicate and interact with others (Hodges et al., 2020). Gastrointestinal disorders are a common comorbidity of autism with a median of 46% of autistic children suffering from GI problems (Holingue et al., 2018). Autistic children are three times more likely to suffer from GI disorders than other children (Wang et al., 2011; Fulceri et al., 2016). The composition of the gut microbiome and metabolome in autistic individuals, is distinct from non-autistic individuals (Li et al., 2019; Mehra et al., 2023). Increases in species belonging to the genera *Bacteriodes* (Finegold et al., 2010; Ahmed et al., 2020)*, Desulfovibrio* (Finegold et al., 2010), *Clostridium* (Finegold et al., 2010; Altieri et al., 2011; Kandeel et al., 2020) and *Ruminococcus* (Ahmed et al., 2020) occur in autistic individuals and possibly contribute to behavioural features; treatment of autistic children with vancomycin improved gastrointestinal symptoms and remarkably their cognitive function (Sandler et al., 2000). Microbiota-derived metabolites [MDM] from these microbiota and numerous others, such as propionic acid (Ossenkopp et al., 2012; Strati et al., 2017) and uremic toxins like p-cresol (hereafter 4-methylphenol [4MP] according to the IUPAC nomenclature) (Altieri et al., 2011; Strati et al., 2017), are hypothesized to aid in the instigation of behavioural features in autistic individuals, through increased host inflammation, particularly in the gut and brain (Peralta-Marzal et al., 2021). Abundances of MDMs are hypothesized to be particularly important in young children when development is rapid, as the effect size can be larger.

Needham et al. (2021), identified 4EP abundance in plasma to be the most significantly differentiated xenobiotic metabolite between autistic and non-autistic individuals, but found no significant differentiation between 4EP abundance in the stool between autistic and non-autistic individuals (Needham et al., 2021). Accordingly, microbial species with the documented functional capacity for 4EP synthesis have not been observed to be enriched in the colon of autistic individuals (Siezen et al., 2009). Therefore, understanding the flux of 4EP within the body, particularly in the context of autistic individuals, is important to identify how small quantities of 4EP may modify neurological development. Evidence of 4EP metabolism, transport, excretion (Xie and Wen, 2020; Lin et al., 2021), uptake by the brain (Needham et al., 2022), and mechanism of effect within the body is limited. This review will outline what is known about the origins of 4EP and its route to, and effects upon, the human brain.

## What is 4EP and where does it come from?

The small phenolic compound, 4-ethylphenol [4EP], is best known as the source of an off-odour in wine vats and other fruit fermentations (Castro et al., 2015). More recently a fascinating role in human physiology has been hinted at with the discovery that high concentrations of 4EP, and its metabolite 4-ethylphenol sulfate [4EPS], in human urine, plasma or stool are associated with neurological and behavioural changes associated with autism (Hsiao et al., 2013; Needham et al., 2021; Campbell et al., 2022) (Table 1).

**TABLE 1:** Overview of clinical studies investigating the relationship between 4EPS and autism.

Study	Main MDM findings	Related phenotype
Needham et al. (2021)	Autistic children had 6.89-fold more 4EPS in plasma than children without	n/a
Campbell et al. (2022)	Oral metabolite-sequestrant reduced 4EPS 4-fold in the plasma and urine of 30 autistic individuals	Improvement in anxiety and irritability following end of treatment period

**FIGURE 1:**
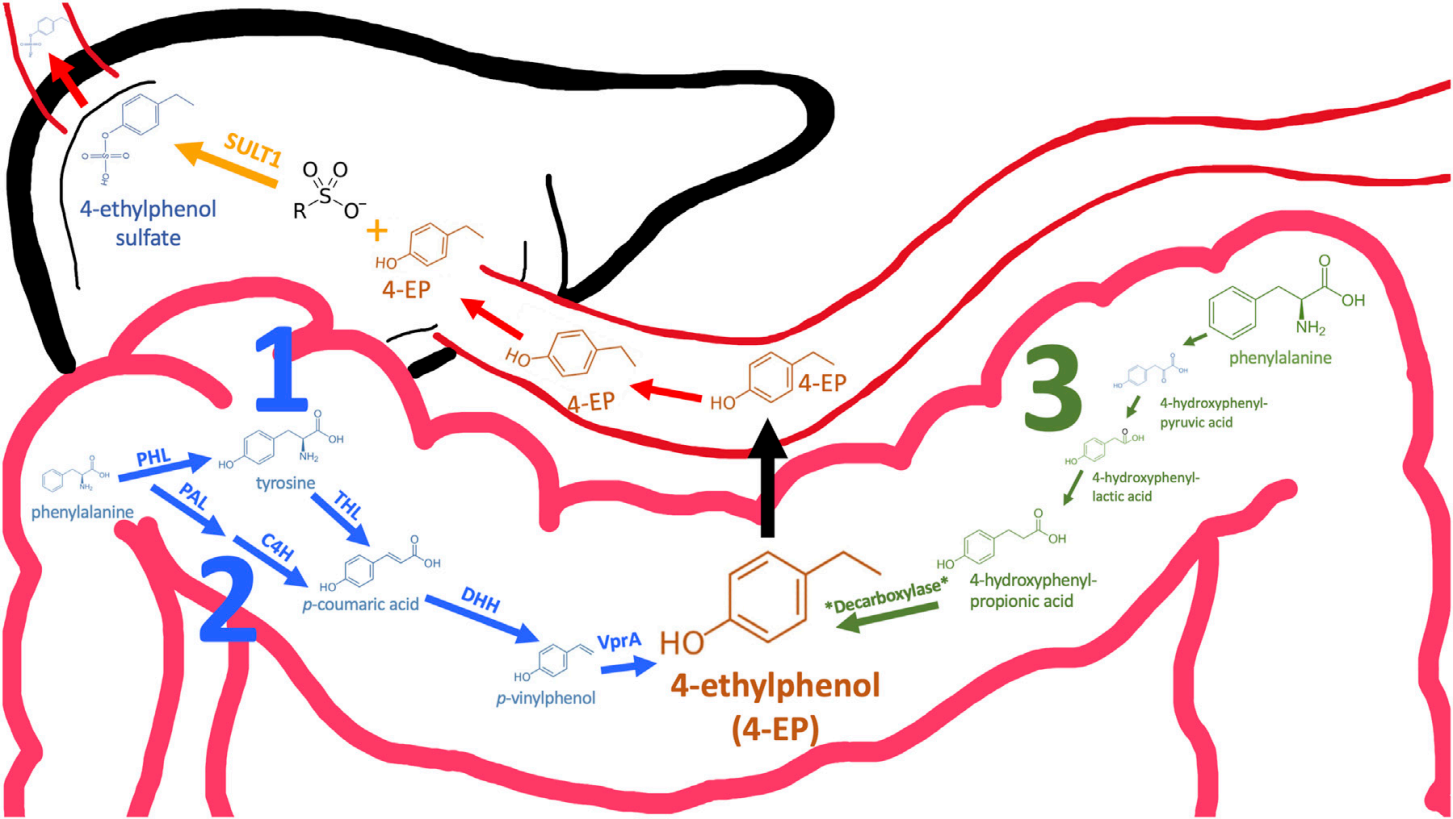
Three pathways may contribute to 4EP formation in the colon. Pathways 1 and 2 converge (coloured blue) and the enzyme names required to catalyse specific reactions are annotated above the arrow. Except for the final reaction (annotated *Decarboxylase*), the pathway in which 4EP may be made in a way similar to p-cresol (pathway 3, green) is well-established (32). Therefore, enzyme and metabolite names have been omitted. Following formation in the colon 4EP crosses the epithelial membrane to the hepatic portal vein via an unknown mechanism. When 4EP enters the liver it is sulphated by sulfotransferase SULT1 to form 4EPS (33). 4EPS subsequently leaves the liver and travels to peripheral tissues through the circulatory system. PAL, Phenylalanine ammonia-lyase; C4H, Cinnamate 4-hydroxylase; PHL, phenylalanine hydroxylase; THL, tyrosine hydroxylase; DHH, decarboxylate hydroxycinnamic acid hydroxylase; VPR, Vinyl phenol reductase (3, 26–28).

4EP is not synthesised by humans but by our microbiome, particularly the gut microbiome (Chatonnet et al., 1992). 4EP is formed by three known pathways, each requiring specific precursor molecules and a series of enzymatic transformations [Figure 1 (Zheng et al., 2020; Nishiyama et al., 2010; Santamaría et al., 2018; Zheng et al., 2021)]. A subset of the required enzymes (e.g., hydroxycinnamate reductase enzymes (HCRs), tyrosine ammonia-lyase (TALs)) are encoded within the genomes of many microbes (e.g., *Clostridium aerotolerans*, *Clostridium xylanolyticum* and *Bacteroides ovatus*) (Chamkha, Garcia, and Labat, 2001; Needham et al., 2022). However, other enzymes (e.g., vinylphenol reductase protein (VPR)) are rarer and encoded by only a few microbes (i.e., *Lactobacillus plantarum, C. aerotolerans DSM 5434T* and *Brettanomyces (Dekkera)* yeasts) (Chamkha, Garcia, and Labat, 2001; Dias et al., 2003; Harris et al., 2009; Santamaría et al., 2018). Notably, even fewer colonise the human gut, and these have not been linked to autism (Siezen et al., 2009). However, Needham et al. (2022) demonstrate that increasing the production of precursors, *p*-coumaric acid and *p-*vinylphenol, albeit via genetic modification, can increase 4EP production (Needham et al., 2022). High abundance of microbes that produce 4EP precursors paired with modest abundances of VPR-producing microbes like *L. plantarum*, may lead to increased 4 EP production. For example, *Clostridium* spp. are highly abundant in autistic individuals and yield 4EP precursors (Finegold et al., 2010; Santamaría et al., 2018).

Another phenolic metabolite, 4MP, is also microbially derived and has a very similar structure to 4EP (Table 2). Hsiao et al. (2013) suggest the structural similarity of 4EP to the predominantly *Clostridioides*-derived 4MP indicates that they are produced by similar biosynthetic pathways (Hsiao et al., 2013). This has been extrapolated to implicate the involvement of *Clostridial* spp. in 4EP production (Finegold, 2002). The close structural similarity between 4MP and 4EP, and 4MPS and 4EPS, suggests they share functional similarities, so in the absence of knowledge of 4EP fluxes and metabolism, we consider the better documented mechanisms of 4MP to be closely analogous. Consistent with this, high plasma and urine concentrations of 4MPS are also associated with anxiety and irritability in autistic individuals (Campbell et al., 2022). 4MP and 4EP tend to be found in their sulphated form in the blood (Morinaga et al., 2004; Martinez et al., 2005; Needham et al., 2022). Notably, 4MPS exists at much higher concentrations than 4EPS (Campbell et al., 2022), however, 4EPS is the more closely correlated metabolite to presentation of autism-like features (Needham et al., 2021) hence we want to focus this review on 4EP. The presence of the extra carbon in 4EPs sidechain seems to induce exceptional potency in its mechanism of effect.

**TABLE 2:** 4EP and 4MP, as well as their sulphated conspecifics 4EPS and 4MPS, share a phenolic core structure, with similar molecular weights [MW], pka and log*P (PubChem-* 31242, 2879*,* 20822573, 4615423, respectively, *HMDB-* HMDB0029306, HMDB0001858, HMDB62551, HMDB11635, respectfully, *ALOGPS, ChemAxon)*. (4-Ethylphenol, 2023; 4-Ethylphenyl Sulfate, 2023; P-Cresol, 2023a; P-Cresol, 2023b; Human Metabolome Database, 2023d; Human Metabolome Database, 2023c; Human Metabolome Database, 2023b; Human Metabolome Database, 2023a; Hansch et al., 1995; Pearce and Simkins, 2011) (4-Ethylphenol, 2023; 4-Ethylphenyl Sulfate, 2023; P-Cresol 2023a; P-Cresol, 2023b; Human Metabolome Database, 2023d; Human Metabolome Database, 2023c; Human Metabolome Database: Showing Metabocard for 4-Ethylphenylsulfate (HMDB0062551)” 2023; Human Metabolome Database, 2023a; Hansch et al., 1995; Pearce and Simkins 2011).

	4EP (4-ethylphenol)	4MP (4-methylphenol)	4EPS (4-ethylphenol sulfate)	4MPS (4-methylphenol sulfate)
	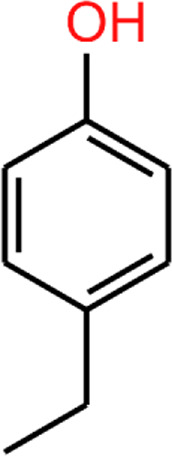	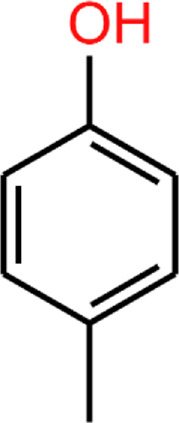	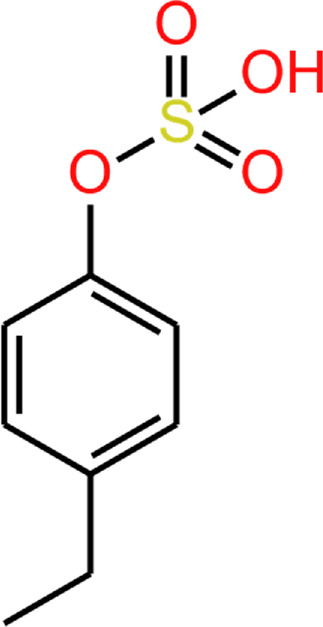	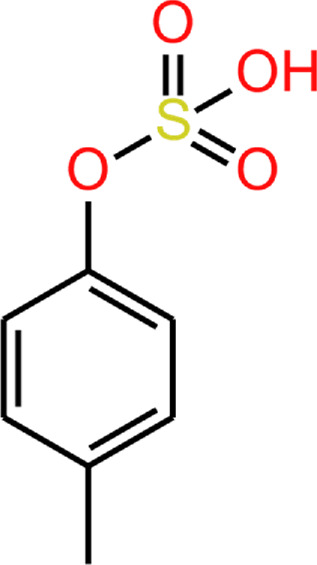
**MW**	122.17	108.14	201.22	188.20
**pKa**	10.00	10.26	−1.90 ^(predicted)^	−2.00 ^(predicted)^
**log*P***	2.58	1.94	2.15/1.7/0.15	1.71/1.3/-0.55

## How does 4EP transit into the blood?

**FIGURE 2:**
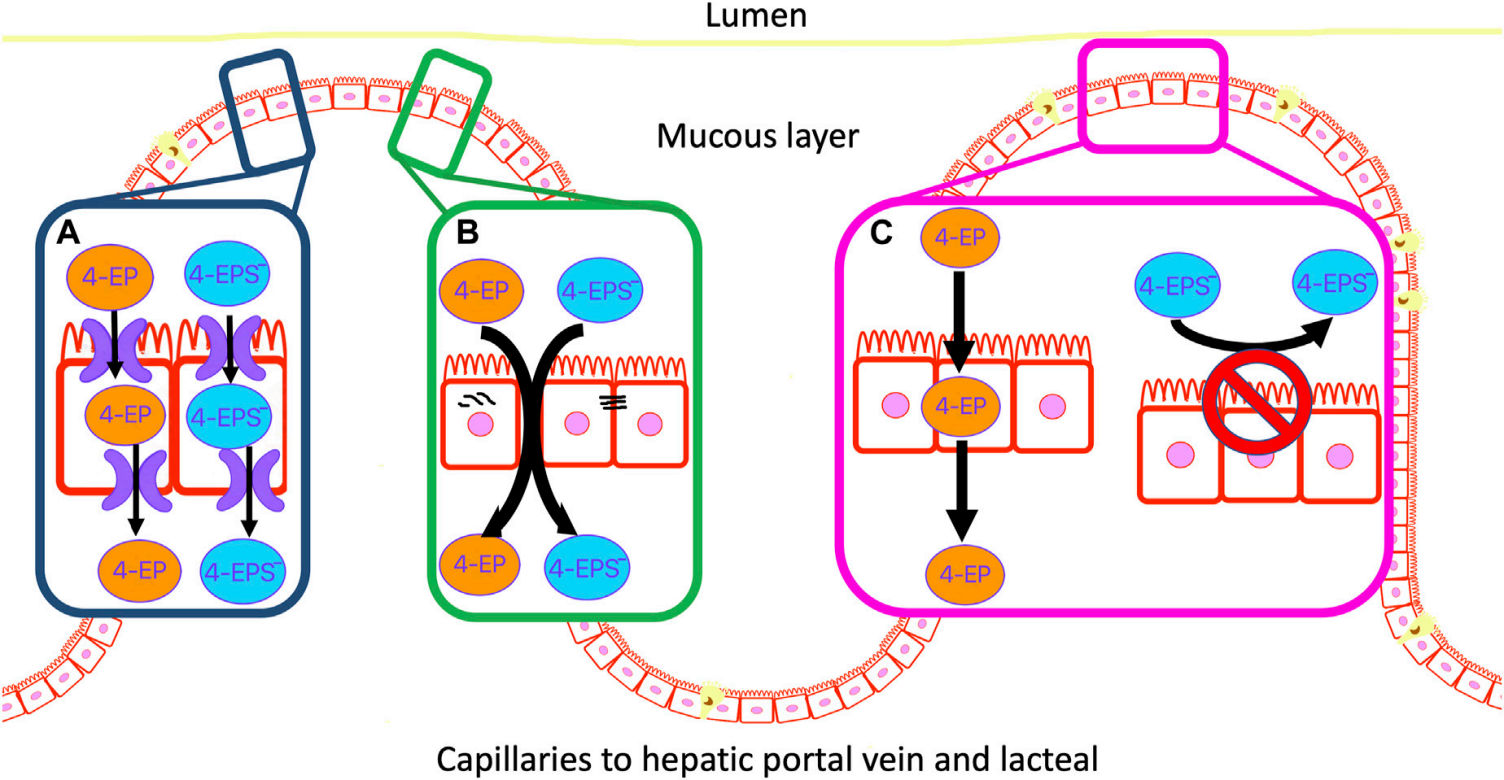
4EP can move through the colon epithelial membrane by three possible mechanisms. **(A)** Active transport of 4EP and/or 4EPS through the membrane. **(B)** Localised mis formation of tight junctions forms paracellular gaps in the membrane and allows 4EP and/or 4EPS to leak through the colon epithelial membrane. **(C)** 4EP passively diffuses through the lipid bilayer due to its small size, relative non-polarity, and lack of charge. 4EPS does not pass through the phospholipid bilayer due to its charged and polar metabolite characteristics.

Cell membranes generally restrict non-specific transcellular transport and those of the gut epithelium are no exception. However, some compounds are able to passively diffuse through cell membranes, depending on their characteristics (e.g., size, solubility, concentration and polarity) (Hussain et al., 2019) (Figure 2). Typically small, hydrophobic and uncharged molecules can passively diffuse through the phospholipid bilayer of the colon membrane (Cooper, 2000). One such small molecule is 4EP (<900 DA) with a molecular weight of just 122.17 DA (Hadacek and Bachmann, 2015; 4-Ethylphenol, 2023). The pH of a healthy adult colon ranges from 5.7–6.7, depending on the location within the colon (Fallingborg, 1999). 4EPs pKa, indicates the pH in which the ionized and unionized forms of a compound exist in equal concentrations, of 10.00 indicates it will be predominantly non-ionised within the colon (Table 2) (Wayne Schultz, 1987). Being in the non-ionised form is expected to increase 4EPs capacity to passively diffuse through the colon membrane. 4EP partition coefficient [log*P*] is 2.58 which also indicates it is non-polar enough to passively cross the colon membrane (Hansch et al., 1995). While active transport remains a possibility, no active transcellular transporters of 4MP or 4EP have been identified in the colon. Therefore, evidence suggests that 4EP passively diffuses through the colon epithelial cells and thus enters the portal vein.

Tight junctions connect colon epithelial cells and restrict paracellular movement of metabolites from the colon into the blood, and *vice-versa* (Hussain et al., 2019). High concentrations of phenols delocalise tight junctions and cause reductions in cell viability in colonic epithelium cell cultures (McCall et al., 2009; Pedersen, Brynskov, and Saermark, 2009). Notably, these studies used phenol metabolites at concentrations that were around one thousand-fold greater than their physiological concentrations. However, incubation of colon HT29 and Caco-2 cell cultures with more physiologically relevant concentrations (0.2, 0.5, 1.5 and 3 mM) of 4MP identified a negative correlation between 4MP concentration and colonocyte viability (Hinai et al., 2019; Smith and Macfarlane, 1996). Notably, cell viability did not decline until >0.5 mM, although 4MP concentrations as low as 0.2 mM were associated with ∼30% colonic cell DNA damage. Physiologically relevant 4EP concentrations in the gut are unknown. Future research should determine whether high concentrations of 4EP affect colonocyte viability and tight junction formation.

Epithelial degradation forms channels through the wall of the colon enabling metabolites to freely move across this barrier and enter the blood. This is commonly known as a “leaky gut” (Camilleri, 2019; Obrenovich et al., 2015) (Figure 2). There is evidence of colon epithelial degradation in autistic individuals, suggesting unregulated trans- and para-cellular transport may enhance 4EP transportation into the blood (Hsiao et al., 2013). However, Needham et al. colonised the gut of germ-free [GF] mice with genetically modified *B. ovatus* that produce excess 4EP precursors, and *L. plantarum* which subsequently produces excess 4EP, yet observed no evidence of dysfunctional intestinal permeability (Needham et al., 2022). Therefore, elevated 4EP concentration is probably not an acute, causal factor in the leaky gut phenotype. However, chronic exposure to 4EP, possibly in combination with other factors, such as aberrant immune regulation and elevated concentrations of other metabolites such as 4MP, may still produce a leaky gut.

## How is 4EP modified in the blood?

Upon crossing the intestinal wall small molecules enter blood vessels that converge with the hepatic portal vein and are carried directly to the liver. Kinetic studies of 4MP metabolism show that, once in circulation, it undergoes rapid sulfation by phenolic sulfotransferase enzymes, SULT1A1/2, which are active in the liver, gastrointestinal tract, brain, skin, lung and kidney (Hinai et al., 2019). 4EP in GF mice colonised with microbiota engineered to produce high concentrations of 4EP, was undetectable in the serum of these mice, whereas 4EPS was abundant, indicating that 4 EP metabolism mirrors that of 4MP (Needham et al., 2022). *In vitro* sulfotransferase assays using cytosolic extracts of mouse tissues, or using recombinant enzymes, showed sulfation of 4EP to 4EPS (Needham et al., 2022). In humans 4MP has been observed to circulate almost exclusively as 4MPS (Martinez et al., 2005). In humans, 4EPS likely dominates too. Sulfation of 4EP to 4EPS has implications on the metabolite characteristics (Table 2), affecting its flux within the body.

Sulfotransferase (SULT) enzymes are a diverse family of enzymes that transfer negatively charged sulphate groups from 3′-phosphoadenosine-5′-phosphosulfate to metabolites (Gamage et al., 2006). In humans, SULT1A1 and 1B1 are found in the liver and colon, while 1E1 is only detected in the liver and small intestine (Teubner et al., 2007). Sulfation is primarily attributed to the liver, although evidence suggests 4EP sulfation during epithelium transcellular passage contributes to 4EPs high sulfation rate (Teubner et al., 2007). The SULT1A1 and 1B1 enzymes are more abundant in the colon compared to the liver in humans (Teubner et al., 2007) and 1A1 is approximately equally abundant in the colon and liver in mice (Needham et al., 2022). Concurrently in mice, sulfation of 4EP in mice liver tissue was highest (below 10^5^), followed closely by colon tissue (above 10^4^) (Needham et al., 2022). Interestingly, mutations in the SULT1A gene locus have been identified in autistic individuals, correlating to low expression of SULT1A in the colon (Hartzell and Seneff, 2012). This may reduce 4EPs rate of sulfation leading to increased circulation in the 4EP form.

Sulfation is one of several metabolic transformations evolved to increase the polarity of mid-and non-polarity molecules, both endogenous and exogenous in origin. It is widely accepted that a purpose of sulfation of metabolites is to facilitate their excretion by the kidney: The increased polarity makes them more water soluble. The effect of sulfation on 4EPS is illustrated by the lower log*P* of 4MPS and 4EPS over their non-sulfated forms (Table 2). Interestingly, alternative metabolic transformations in this pathway, such as glucuronidation, produces metabolites that are more polar, yet are produced in lower amounts. For example, 4EP-glucuronide has estimated log*P* values of 0.8, 0.03 and 0.68: a mean value 0.83 units lower than 4EPS. 4EP-glucuronide is present at approximately 10% of the concentration of 4EPS in healthy individuals (Meert et al., 2012).

The kidney is a key organ in the excretion of small phenolics, such as 4MPS and 4EPS (Bush et al., 2017; Wu, Bush, and Nigam, 2017). People with chronic kidney disease have 4MPS concentrations 10 times greater than that of healthy controls and hemodiafiltration only removed 37% of 4MPS (Meert et al., 2012). Several studies claimed that >90% of 4MPS in circulation is bound to proteins such as albumin, preventing its excretion by the kidneys (Kikuchi et al., 2010; Caraceni, Tufoni, and Bonavita, 2013; Poesen et al., 2016). However, Bergé-Lefranc et al. (2010) calculated that only 20% of 4MPS was present in circulation bound to albumin and concluded that protein binding was not sufficient to explain why 4MP and other uremic toxins were not removed by haemodialysis (Weisiger, 1985; Bergé-Lefranc et al., 2010). The direct association of 4EPS with albumin is yet to be measured, although 4EPS is also identified as a uremic toxin in a rat model of chronic kidney disease (Velenosi et al., 2016). Compromised renal function is observed in >25% of autistic adults and other individuals with neuro-atypical phenotypes (Clothier and Absoud, 2021). Hindered excretion of 4EPS is a possible mechanism of 4EPS accumulation in the blood of autistic individuals (Needham et al., 2021).

Other mechanisms which may contribute to the persistence of 4EP in the human body include variations in the rate of other metabolic transformations which target the ethyl chain. Metabolic simulations suggest that the enzyme Cytochrome P450 1A2 can convert the ethyl chain into vinyl, methyl ketone, or hydroxylated products, all of which are more polar (Djoumbou-Feunang et al., 2019). The ethyl chain is the part of 4EP which makes it amphipathic, and capable of binding to non-polar surfaces or ligands, such as albumin. Modification of this part of 4EP may be a critical property in reducing such binding and facilitating its excretion. Crucially, a study comparing alleles of Single Nucleotide Polymorphisms with suppressive effects on the metabolic activity of Cytochrome P450 1A2 between a small group of autistic children and a non-autistic cohort identified three that are significantly correlated (Miot et al., 2019).

## How does 4EPS get into the brain?

**FIGURE 3:**
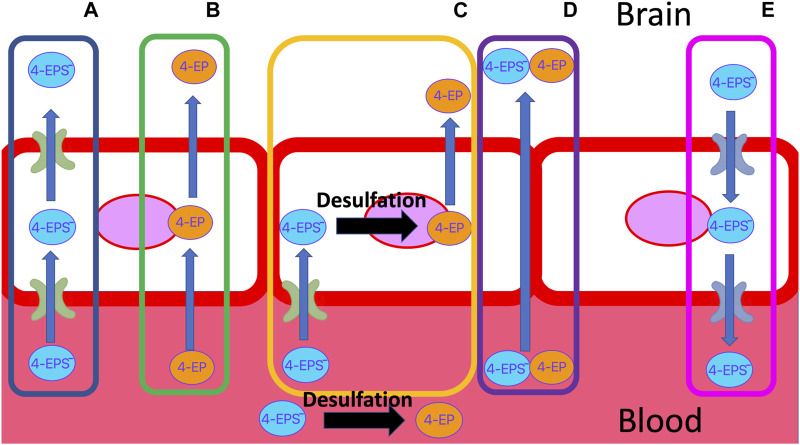
Possible transport mechanisms of 4EP and negatively charged 4EPS, through the BBB. **(A)** 4EPS is actively transported through the apical and basolateral membrane. **(B)** 4EP passively diffuses through the lipid bilayer due to its small size, solubility and lack of charge. **(C)** 4EPS is transported into the BBB epithelial cell through an influx transporter. Once in the cell, 4EPS is desulfated to 4EP, which can then passively diffuse into the brain. **(D)** Delocalised tight junction formation results in paracellular gaps in the membrane that allow 4EP and/or 4EPS to pass through the barrier. **(E)** Efflux transporters actively transport 4EPS out of the brain, e.g., OATs. BBB, blood brain barrier.

Accumulation of 4EPS in the brain is linked to aberrant demyelination and reduced connectivity between important regions of the brain (Needham et al., 2022). How 4EPS crosses the BBB is unknown. The BBB is a layer of endothelial cells with similar properties to the intestinal wall (Daneman and Rescigno, 2009; Obrenovich, 2018), but with additional restrictions on passive diffusion (Gloor et al., 2001; Daneman and Rescigno, 2009). Small (<500 DA), non-ionised (4< pKa<10) compounds that are mid polarity (log*P >2* = 2–4) are moderately water soluble but also lipophobic enough to pass through the membrane of the BBB passively (Obrenovich, 2018; Fischer et al., 1998; Lipinski et al., 2001) (Figure 3). Sulfation of 4 EP to 4EPS increases the molecular weight from 122.17 DA to 201.22 DA, reduces the pKa from 10.00 to a calculated −1.90, and log*P* from 2.58 to 0.15 (Table 2). Thus, 4EPS is less likely to pass through the BBB, when compared to 4EP. Notably, dosing mice with structurally similar 4MP (2.5 mL/kg) via a gastric tube, results in a flux of 4MP and 4MPS into the blood to various organs (Morinaga et al., 2004). Equal concentrations of 4MP are measured in the brain and blood, but 4MPS was found in the brain at 10%–20% of the blood concentration after 4 h (Morinaga et al., 2004). This supports 4MP and 4MPS having different abilities to cross the BBB and suggests that there is sulfotransferase activity inside the BBB (Figure 3).

**FIGURE 4:**
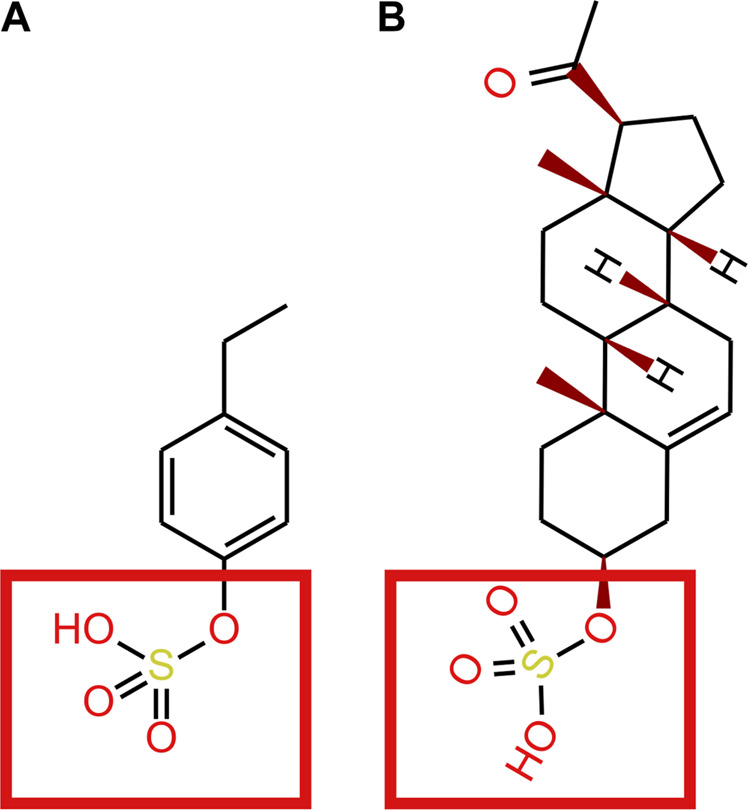
**(A)** 4EP **(B)** pregnenolone sulphate. Red squares show where the STS enzyme binds to pregnenolone sulphate and where we suggest an STS enzyme may bind to 4EPS to desulfate to 4EP.

Sulfatase enzymes remove sulfate groups from compounds. Enzymes such as steroid sulfatase are known to be essential to brain function (Kříž et al., 2008; Roberts et al., 2017; Cardona et al., 2019; Morimoto et al., 2022). Sulfatase activity in endothelial BBB cells deconjugate the steroid, pregnenolone sulphate, to its free form (Qaiser et al., 2017). Sulfatase enzymes that deconjugate 4EPS have not been identified. Although structural similarities between where the sulfate group attaches to steroids and 4EPS suggest the active site on steroid sulfatase’s may be capable of binding to 4EPS (Figure 4). As such, it is possible 4EPS is desulphated back to 4EP in the endothelial BBB cells, which can passively enter the brain (Figure 3). However, a mechanism for 4EPS (Figure 4) transport through the apical membrane of the BBB is still unknown and the potential for steroid sulfatase enzymes to act on 4EPS should be tested.

**FIGURE 5:**
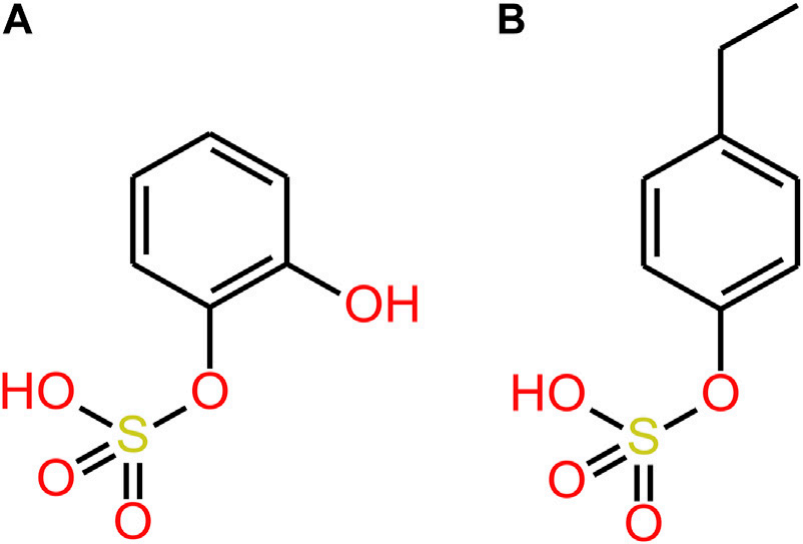
**(A)** Catechol-o-sulfate **(B)** 4EPS.

Transporters of the endothelial BBB may transport 4MPS and 4EPS into the BBB (Figure 3). OAT3 transports 4EPS through the BBB in mice. However, OAT3 is an effluent transporter and the fact that it transfers 4EPS across the BBB does not explain how 4EPS enters the brain (Hosoya and Tachikawa, 2011). *In vitro* studies assessing the ability of small phenolics (e.g., Pyrogallol-O-sulfate, Catechol-O-sulfate, 1-O-methylpyrogallol-O-sulfate, 4-Methylcatechol O-sulfate) to cross the BBB, demonstrated that sulfation of compounds, specifically catechol-O-sulfate and pyrogallol-O-sulfate, enhanced their ability to cross the endothelial cell membranes despite *in vitro* analyses suggesting against their passive BBB permeation ability (Figueira et al., 2017). Figueira et al. hypothesised that sulfation enhances active transport, however, no specific transporters were identified. Catechol-O-sulfate and 4EPS are structurally similar (Figure 5), suggesting active transporters aid in 4EPS crossing the BBB. In humans there are a family of 10 transmembrane OAT proteins that are a subfamily of the solute carrier 22 transporters (Nigam et al., 2015). The functionality of the human OATs within the BBB remains largely unidentified, in particular their specificity for and directions of microbial-derived metabolite transport (Schäfer, Meyer Zu Schwabedissen, and Grube, 2021). Further research should focus on identifying BBB influx transporters that promote the movement of 4EPS into the brain.

BBB integrity is characteristically reduced in autism (Fiorentino et al., 2016; Obrenovich et al., 2015; Obrenovich, 2018) and it is possible that 4EPS leaks into the brain through channels that are like those formed in the intestinal wall of a leaky gut (Fiorentino et al., 2016) (Figure 3). Moreover, it is possible that 4EPS contributes to the degradation of tight junctions within the BBB and that these para cellular channels facilitate crossing the BBB.

## How does 4EPS affect the brain?

4EPS concentrations have been linked to neurological disorders (Hsiao et al., 2013; Needham et al., 2021; Campbell et al., 2022). Systemic administration of 4EP induced anxiety-like behaviours in mice (Hsiao et al., 2013; Hsiao et al., 2013). These findings have been reproduced in a recent mouse study that colonised microbiomes of GF mice with modified *B. Ovatus* and *L. plantarum* that produce high concentrations of 4EP (Needham et al., 2022).

It is unclear whether there is a difference in toxicity between 4EP and 4EPS. The literature often assumes 4EPS has greater negative effects in the body than 4EP, although no evidence exists to support this assumption (Velenosi et al., 2016; Needham et al., 2021; Campbell et al., 2022). Adding further ambiguity to the situation, 4EP and 4EPS are often used interchangeably in the literature. Thus, the relative contributions of 4EP and 4EPS to autism, neurological conditions and anxiety-inducing phenotypes is even more challenging to untangle (Takahashi et al., 2006; Velenosi et al., 2016; Needham et al., 2022). For example, Needham et al. (2022) discriminate between 4EP and 4EPS, whereas Needham et al. (2021) quantified 4EPS only (Needham et al., 2021; 2022). Discriminating between 4EP and 4EPS is essential to distinguish their relative effects on *in vitro* immune response assays in brain tissues, and their flux within the body. Time-series flux experiments have been completed for 4MP (Morinaga et al., 2004), but differences in molecular characteristics mean that these results cannot be extrapolated directly to 4EP pharmacokinetics. Understanding 4EP pharmacokinetics is important as temporal changes in the relative ratio of 4EP to 4EPS in the brain suggests the mechanism of entry. For example, a high initial abundance of 4EP relative to 4EPS may indicate a process of desulfation in the BBB, whereas a high initial abundance of 4EPS suggests either active transport or a “leaky” BBB.

Needham et al. (2022) found that 4EPS accumulation in the brain impacted the functional connectivity between subregions of the hippocampus, thalamus, amygdala, hypothalamus, piriform and cortex (Needham et al., 2022). This accumulation of 4EPS was associated with a significant increase in glucose uptake (i.e., activity) in the subregions of the hypothalamus, amygdala, the bed nucleus of stria terminalis and the paraventricular nucleus of the thalamus (Needham et al., 2022). Notably, these brain regions have inter-related functions that include controlling anxiety behaviours and responses to stress stimuli (Goode et al., 2019; Forster et al., 2012; Fischer, 2021; Barson, Mack, and Gao, 2020).

The presence of excess (>15 nM) 4EPS in the brain, is associated with significant downregulation of *Oligodendrocyte myelination glycoprotein* and *Opalin* genes (Needham et al., 2022). Simultaneously, excess 4EPS associates with upregulation of non-myelinating oligodendrocyte progenitor cells (Needham et al., 2022). These changes were accompanied by reductions in myelination of neuronal axons. Efficiency losses in neural conductivity resulted in reductions in controlled neuron pathways and connectivity (Needham et al., 2022). Therefore, acute exposure to high concentrations of 4EPS degrade neuronal communication (Needham et al., 2022). Levels of 4EPS in healthy mouse brains are below the limit of detection so the effects observed by Needham et al. may not be representative of real-world exposure. It is not understood whether chronic exposure to moderate 4EPS levels result in cumulative damage with similar effects. Future work should determine if this is the case using long term *in vitro* (human), and *in vivo* (mice) experiments. No measurements have been made in human brains.

## Future directions

Reducing the levels of 4EP at the source, i.e., in the colon, may be a viable mechanism to limit the damaging effects of 4EP. Theoretically these reductions can be achieved by a) reducing production at the source, b) reducing bioavailability, and c) promoting removal from the body. Of these options, a) and b) have currently been demonstrated as viable. For example, reductions of production at source, which correlate with alterations of the gut microbiome, have been achieved using faecal microbiota transplants, vancomycin treatment, and dimethyl fumarate treatment in autistic individuals and multiple sclerosis patients, respectively (Kang et al., 2020; Ntranos et al., 2022; Sandler et al., 2000). This is an active area of research with multiple clinical trials currently underway (e.g., NCT04246398, NCT04878718, NCT04630847, NCT04948814, NCT03408886; (ClinicalTrials.Gov, 2023) Sequestration of 4 EP, has also been demonstrated to be a viable method for the manipulation of bioavailability. For example, AST-120, a high-surface-area spherical carbon adsorbent with an affinity for uremic toxins, can be used to remove 4MPS and 4EPS from the gut reducing its circulating concentrations by approximately 4-fold (Campbell et al., 2022).

Interestingly, high abundances of 4 MP in the urine of 2–8-year-old autistic children is positively correlated with chronic constipation, while no associations were found between the presence of “4MP-producing” *Clostridium* spp. in the gut flora (*p* = 0.92) or augmented intestinal permeability (*p* = 0.18) (Gabriele et al., 2016). However, a follow-up 6-month prospective study in 21 autistic children found inconsistencies in this correlation (Turriziani et al., 2022). It is unknown if 4MP and 4EPs production is correlated, so this link is not direct evidence of 4EPs correlation to stool transit time. In future, a comparison of the abundances of 4EP in the stool, urine and blood, of constipated autistic individuals before and after constipation treatment will clarify if stool transit time affects 4EPs production and accumulation in the body.

When considering the roles of microbes in the gut microbiome, it is important to recognise that VprA production, the rate-limiting enzyme in the production of 4EP, in species is insufficient to support general conclusions about their effect on human health. Rather, these findings are correlative until empirically proven by intervention trials. For example, *L. plantarum* is known to express the specific VprA enzyme required for 4EP formation (Santamaría et al., 2018). Yet, *L. plantarum* is incorporated into some commercial probiotics because it has beneficial effects that include a reduction in anxiety (Liu et al., 2019). The strain of *L. plantarum* probiotics that improved autism phenotypes in autistic boys encodes copies of the VprA gene (LBHS01000000, 2023; Liu et al., 2019; Liu et al., 2019). Conversely, it has been shown that *B. ovatus,* one of many microbes capable of converting tyrosine to 4EP-precursor, *p*-coumaric, competes with the host for vitamin B12, aggravating B12 deficiency. This is important as B12 administration improves metabolic abnormalities in autistic individuals along with clinical symptoms (Rossignol and Frye, 2021). *B. ovatus* sequesters B12 from the host via btuB upregulation and ATP production promoting energy-dependant translocation of vitamin B12 transporters at the inner membrane, enhancing *B. ovatus* colonisation in the gut (Chen et al., 2023). Increased *B. ovatus* may impact autistic individuals through aiding production of neurotoxins and reducing beneficial metabolites (e.g., B12). These correlations demonstrate the need to employ empirical studies to better understand the complex associations of microbes with neurological health. Such studies, must incorporate methods that enable an understanding of the cumulative functions of a single microbe as well as its complex interactions with other microbes.

Understanding the putative associations between 4EP and 4EPS and neurological status (e.g., Autism) requires that 4EP and its metabolites are quantified in the human brain. Whilst brain biopsies are the gold standard, they are invasive, which hinders their use in research. Analyses on pre-existing or post-mortem brain samples are possible, although the utility of these samples for studying the dynamics of 4EP and its related compounds is limited. Cerebral spinal fluid is more easily accessible, although cerebral spinal fluid is only a proxy for the brain. Despite the difficulty associated with obtaining “appropriate” samples, it is essential to compare 4EP levels in autistic and non-autistic individuals. This comparison will enable us to determine if the higher blood and urine abundance of 4EPS in autistic individuals correlates with an increased abundance in the brain. Furthermore, animal and organoid studies will enable the identification of the brain cell-types that show the greatest response to 4EPS exposure. This will enable the development of time-course targeted metabolomic experiments, using isotopically labelled 4EP, that detect the metabolic activity of 4EP within the cell. Such, targeted experiments will provide mechanistic insights that aid the discovery of 4EPs relationship to anxious behaviours.

How 4EP moves into the body and brain is critical to understand. Currently, there is a lack of evidence to support the relative contributions of passive and active transport mechanisms to 4EP movement across the intestinal or BBB lipid bilayers. This precludes the targeted design of future treatments to inhibit 4EP transport into the body or brain. Mice exposure time-series studies to isotopically labelled 4EP (e.g., (Morinaga et al., 2004) will enable the untargeted measurement of 4EP and 4EPs flux within the body and tissues. Relative concentrations of isotopically labelled 4EP and 4EPS in the brain and blood will confirm the forms in which 4EP crosses the BBB and if it becomes sulfated in the brain. If 4EPS is the immediate dominant form observed in the brain, then the role of active transporters (e.g., OATs) should be further explored using targeted inactivation (e.g., Probenecid and taurocholate inhibition of OAT3 transporters in mice brains identified OAT3 as an important efflux transporter for dehydroepiandrosterone sulfate) (Miyajima et al., 2011). The identification of inhibitors that prevent 4EPSs active transport in the brain, and identification of mechanisms to upregulate brain specific efflux transporters (e.g., OAT3) would enable 4EPS targeted removal from the brain, to reduce its contact time and toxic effects (Hosoya and Tachikawa, 2011).

Promoting the removal of 4EP from the body by driving its metabolism to increase excretion of soluble forms is theoretically possible. For example, the glucuronidated form of 4MP has reduced immune activating and inflammatory effects, when compared to 4MP and 4MPS (Meert et al., 2012; Zhu, Rong, and Kiang, 2021)*.* Notably, only 8%–24% of 4-methylphenol glucuronide is protein bound in the blood, hence 4-methylphenol glucuronide is filtered out of the blood more easily, with 79% being removed by hemofiltration in chronic kidney disease patients (Meert et al., 2012; Poesen et al., 2016). Therefore, glucuronidation represents a possible mechanism by which removal may be promoted. While the percentage of 4EP that normally undergoes glucuronidation is unknown, this should be established. If the glucuronidation of 4EP can be promoted, it would provide a mechanism through which 4EP can be diverted away from 4EP sulfation, and thus may be a possible therapeutic mechanism for removal of 4EP (via 4EP-glucuronide) from the body.

Throughout this review we have referenced many studies that focus on 4MP, as this molecule shares structural and functional similarities to 4EP but has been researched extensively in comparison. We discussed only 4MP flux within the body in this paper, as the sole purpose of including this molecule is to infer possible mechanisms of 4EP flux within the body. To this point, it is essential that research using 4EP is undertaken to understand its specific flux and metabolism.

We decided to research 4EP over 4MP, as although both are correlated to autism (Kang et al., 2018; Gabriele et al., 2016; Altieri et al., 2011; Needham et al., 2021), recent findings suggest 4EP to be a more potent neuromodulator (Needham et al., 2021). Needham et al. (2021) identified 4EP to be the most significantly different xenobiotic metabolite in plasma between autistic (*n* = 130) and non-autistic (*n* = 101) individuals, while 4MP was not significantly different between the groups in the plasma and was surprisingly lower in the stool (Needham et al., 2021). Furthermore, absolute concentrations of 4EP (range of 2–270 μg/L) are much lower than 4MP (range of 550–24,000 μg/L) (Campbell et al., 2022), suggesting the extra carbon has a significant differential effect on flux into the brain or increased action in neurodevelopmental pathways. However, few clinical studies have quantified both these metabolites (Table 1), and further research is needed to clarify if 4EP is indeed a more potent neuromodulator than 4MP. Follow-up studies to understand crucial mechanistic differences between these metabolites would be useful to decipher how differences in potency may originate.

## Conclusion

4EP contributes to neurodegeneration and enhances anxiety-like and autistic phenotypes (Campbell et al., 2022; Needham et al., 2022). The absence of 4EP in GF-mice and evidence that *B. ovatus* and *L. Plantarum* produce 4EP from tyrosine supports a microbial origin for 4EP (Hsiao et al., 2013; Han et al., 2018; Needham et al., 2022). High circulating and tissue concentrations of 4EP (e.g., in autistic people) may occur as a consequence of “leaky guts”, rather than a direct effect of overproduction by gut microbes. Collectively the evidence indicates that 4EP is a highly transportable neuromodulatory microbial metabolite with variable, concentration dependent effects. However, much of the evidence for the mechanisms that affect 4EP accumulation and transport is theoretical, or is extrapolated from 4MP, which has similar properties but a different chemical structure. Identifying factors that affect 4EP bioavailability and transportation in the human body should be a priority for understanding the impact(s) of 4EP on neurological development.

## Abbreviations

BBB, blood brain barrier; C4H, cinnamate-4-hydroxylase; DHH, decarboxylase hydroxycinnamic acid hydroxylase; HCR, hydroxycinnamate reductase enzymes; MW, molecular weight; OAT, organic anion transporter; PHL, phenylalanine hydroxylase; PAL, phenylalanine ammonia-lyase; SULT, sulfotransferase; THL, tyrosine hydroxylase; TAL, tyrosine ammonia-lyase; VPR, vinyl phenol reductase; 4EP, 4-ethylphenol; 4EPS, 4-ethylphenol sulfate; 4MP, 4-methylphenol; 4MPS, 4-methylphenol sulfate.
